# A Case Study on the Development of a High-Intensity Interval Training Set for a National-Level Middle-Distance Swimmer: The Conception of the Faster-than-Race Pace Test Set

**DOI:** 10.3390/jfmk10030291

**Published:** 2025-07-29

**Authors:** Konstantinos Papadimitriou, Sousana K. Papadopoulou, Evmorfia Psara, Constantinos Giaginis

**Affiliations:** 1 Department of Nutritional Sciences and Dietetics, School of Health Sciences, International Hellenic University, 57001 Thessaloniki, Greece; kpapadimitriou@ihu.gr; 2 Faculty of Sport Sciences & Physical Education, Metropolitan College, University of East London, 57001 Thessaloniki, Greece; 3 Department of Food Science and Nutrition, School of Environment, University of Aegean, 81100 Myrina, Greece

**Keywords:** swimming, performance, progression, races, improvement

## Abstract

**Background:** Swimming coaches search for the most efficient training approach and stimuli for swimmers’ improvement. High-intensity interval training (HIIT) is a well-established training approach used by coaches to accelerate swimmers’ improvement. A HIIT variation, which has lately been discussed by many coaches about its possible effectiveness on performance, is Ultra Short Race Pace Training (USRPT). The present case study aimed to examine the effect of a faster-than-race pace test set (FRPtS) on the performance of a middle-distance (MD) swimmer at the freestyle events. **Methods:** This case study included a 21-year-old national-level MD swimmer with 16 years of swimming experience. The swimmer followed 11 weeks of FRPtS sets in a 17-week training intervention. The FRPtS sets were repeated two to three times per week, the volume ranged from 200 m to 1200 m, and the distances that were used were 25 m, 50 m, and 100 m at a faster pace than the 400 m. Descriptive statistics were implemented, recording the average with standard deviation (number in parentheses), the sum, and the percentages (%). **Results:** According to the results, the swimmer improved his personal best (PB) and season best (SB) performance in the events of 200 m and 400 m freestyle. Specifically, the improvement from his PB performance was 2.9% (−3.49 s) and 1.0% (−2.55 s), whereas in his SB performance it was 2.9% (−3.53 s) and 4.4% (−11.43 s) for the 200 and 400 m freestyle, respectively. **Conclusions:** Concluding, FRPtS is assumed to have beneficial effects on the swimming performance of MD events. However, further crossover or parallel studies on different swimming events with more participants and biomarkers must be conducted to clarify the effects of that kind of training on swimming performance.

## 1. Introduction

Swimming performance enhancement requires the implementation of both aerobic and anaerobic (alactic and lactic) energy systems [1,2], as well as technical proficiency [3,4]. To evaluate a swimmers’ training status before, during, and after sessions, various physiological indicators are commonly used, including heart rate (HR) [5,6], oxygen consumption (VO_2_, VO_2_peak, VO_2_max) [7], blood lactate (BL) [8], blood glucose (BG) concentration [9], and ratings of perceived exertion (RPE) [10].

One of the most implemented methods for influencing these physiological indices is high-intensity interval training (HIIT). Coaches widely use this training method to improve swimmers’ performance [11,12,13,14,15,16,17], either as an alternative to or in combination with low- or moderate-intensity continuous or intermittent training, which has been shown to induce fewer physiological adaptations than HIIT [14]. As a result, many studies have investigated the effects of HIIT in swimming [11,14,15,17].

Another often discussed training method is ultra-short race pace training (USRPT) [18], which appears to be a submaximal variation of HIIT with low BL relevance to swimming events [9]. USRPT protocols that were utilized in the experimental studies were 20 × 25 m on freestyle, with a 40 s interval working at a 100 m pace [9]; 20 × 50 m on freestyle, with a 1:1 interval working at a 200 m pace [19]; and 20 × 25 m on freestyle, with a 35 s interval working at a 100 m pace [20]. Although unofficially published data regard its daily implementation [18], it is discouraged due to potential psychological strain [21]. Also, there is much confusion regarding its volume, intensity, and interval, especially in middle-distance (MD) events (200–400 m) [21,22].

MD events are based on work in the aerobic endurance training zone, which obtains high percentages of the total training volume since it is used for warming up and cooling down in each training session, as well as for promoting recovery between intense bouts [23]. Aerobic capacity implies increasing the intensity of the training sets, using the intensive interval training method, and in the competitive period, breaking the events into splits by employing the fractioned race pace training (RPT) strategy (e.g., 15 × 100 m at 1500 m pace with 10 s intervals) [19,24].

The aerobic capacity training zone aims to increase the transport, diffusion, and peripheral perfusion of O_2_, as well as the mitochondrial capacity [25]. The depletion of the muscle glycogen stores will not allow the training series to go over 10 min of duration if conducted at VO_2max_ velocity (vV˙O_2max_) pace (as it should) [25]. In the competitive period, the 400 m pace can be trained by making 4 × 100 m (10 s rest) at race pace (or even faster). Additionally, Papadimitriou [21], in his study, suggested a table with probable sets for MD and long-distance (LD) events (≥800 m) (Table 1 [21]).

Eventually, there are many theories and equivocal information regarding which type of set would probably be more beneficial for MD swimmers’ improvement. HIIT variations such as USRPT have not been examined in depth. However, its implementation for MD or even LD swimmers can probably be imprecise with a low physiological burden. This case study aims to implement a modified USRPT set, called by the authors the faster-than-race-pace test set (FRPtS), which will contribute to MD swimmers’ training at speeds faster than their race pace. This approach familiarizes them with the required swimming speed and physiological demands of their next performance goal. We hypothesize that this variation will contribute to the improvement of MD events performance, utilizing a test set that will enhance swimmers’ interest, and it will speed up the rate of improvement.

## 2. Methodology

### 2.1. Participant

A 21-year-old male, National-level, MD freestyle swimmer (200 and 400 m) with a training experience of 8 years in the racing category and 617 and 650 WA points for 200 and 400 m freestyle, respectively [WA], participated in the study. The swimmer was selected considering his participation in the National Championship and his ability to swim MD events (200–400 m). His personal best (PB) performance was for 200 m, 2:00.50 from 12 January 2019, and for 400 m, 4:14.03 from 3 February 2017.

Also the swimmer’s anthropometrics and body composition(height of 179 cm, weight of 82 kg, free fat mass (FFM) of 76 kg, fat mass (FM) of 6 kg, and body mass index (BMI) of 25.5 kg·m^2^) were recorded. The swimmer underwent training six days a week for approximately three hours, comprising ten sessions (seven focused on swimming and three on dryland training), covering an average weekly distance of 28,224 (5130) m. The dryland training focused on isotonic, isometric and elastic strength exercises.

This case study had a retrospective longitudinal observational design [26] and was carried out from February 2021 to June 2021, the month of the National Championship. Therefore, for the utilization of the training data, written informed consent was obtained from the athlete, who signed a statement permitting the publication of his training data [27] in accordance with the principles outlined in the Declaration of Helsinki (1975, revised in 2013), whereas the Institutional Review Board decision was received for the publication of the descriptive data, ensuring the anonymity considering the participant’s information (Collaborative Research Ethics Committee (CREC), approval code: 494/2025, approval date: 18 June 2025).

### 2.2. Study Design

#### 2.2.1. Periodization

**Figure 1:**
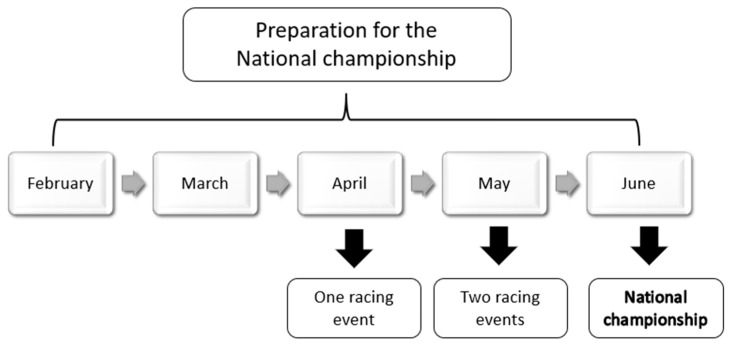
Season plan representation.

During the examined months (February to June), the total number of training sessions per month, the races that he participated in, and the average daily, weekly, and monthly training volume were recorded. Figure 1 presents the periods and the target racing events (Figure 1).

#### 2.2.2. Training Intensity Distribution (TID)

Considering the intensity, the training was split into seven zones, considering his HR in 10 ss [28]. The idea was implemented by the Urbanchek’s color system, which is widely used in collegiate swimming [29]; however, it was implemented in a modified version by the coach (Table 2).

According to the modified version, aerobic zones were split into three subcategories (Z1a, Z1b, and Z1c) for the precise implementation of aerobic training, considering the period. Specifically, Z1a was utilized at the beginning of the training period and the day after a Z3 or Z4 set. Z1b and c were utilized synergistically through progressive aerobic sets in different volumes and intervals. Also, VO_2max_ sets were split into FRPtS and specific racing sets (RS) (i.e., 200 and after 30 min, a 100 m race). In Table 3, an example set is provided for each zone (Table 3).

However, for a more applied approach for the swimmer, the TID was structured as follows: Z1a, Z1b, and Z1c were grouped as Z1; Z2 was treated as a distinct “threshold” category; and Z3, Z4, and Z5 were combined into a single Z3 category representing anaerobic (both lactic and alactic) efforts. The weight of the zones was considered according to the volume in each zone (TIDv).

#### 2.2.3. Faster-than-Race Pace Test Set (FRPtS)

The FRPtS was utilized in four out of five months of the preparation period (from March to June). The total volume of the set in each session varied from 200 (tapering period) to 1200 m (high-load preparation period, with occasionally a combination of sets for 200 and 400 m). Also, the most implemented distances were the 50 and the 100 m for the improvement of 200 and 400 m, respectively. The interval between 50 and 100 m repetitions was determined considering the swimmer’s ability to swim with consistency faster than his race pace and the concepts of USRPT and RPT. Therefore, the interval for the 50 m sets (faster pace than the 200 m event) was close to 20 s, whereas for the 100 m sets (faster pace than the 400 m event) it was 1 min.

### 2.3. Statistics

Descriptive statistics were implemented, recording the average with standard deviation (number in parentheses), the sum, and the percentages (%). The analysis was conducted in SPSS (IBM Corp. Released 2017. IBM SPSS Statistics for Windows, Version 25.0. Armonk, NY, USA: IBM Corp.).

## 3. Results

### 3.1. Sessions

**Figure 2:**
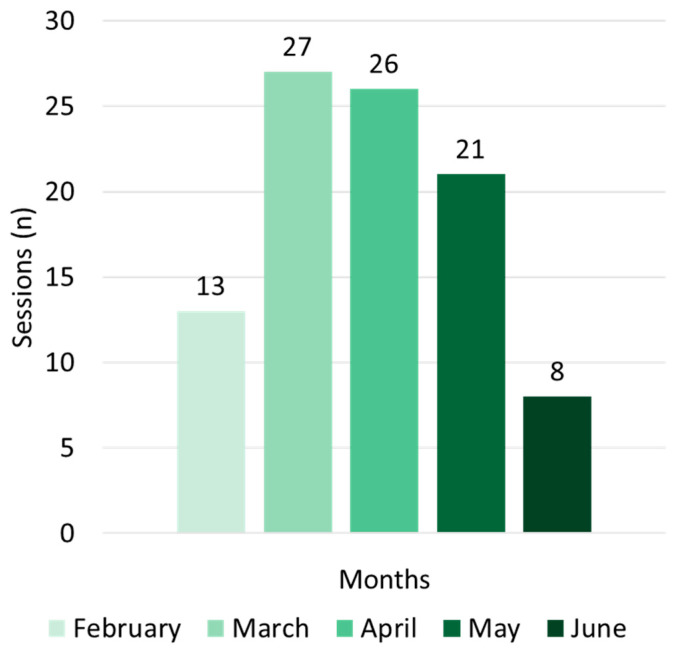
Number of sessions.

The total number of sessions varied from 8 to 27, depending on the period (induction, preparation, or tapering) (Figure 2).

### 3.2. Total Training Volume

**Figure 3:**
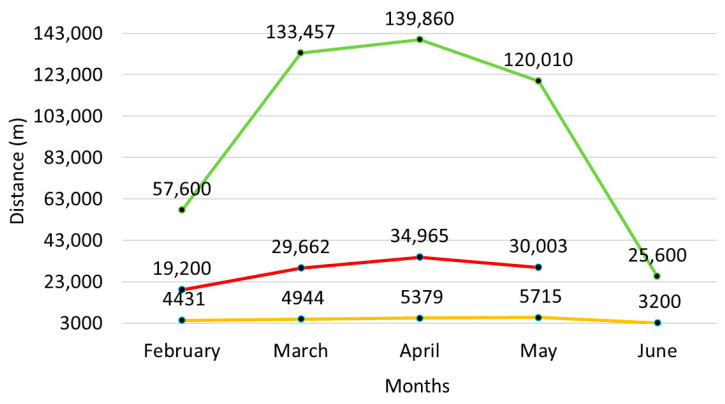
Depiction of the total volume per month (green line), per week (red line), and per session (yellow line).

The swimmer implemented an average of 95,183 (45,359) m per month, 28,224 (5130) m per week, and 4729 (878) m per session. Figure 3 depicts analytically the total volume for each month, week, and session.

### 3.3. TID

**Figure 4:**
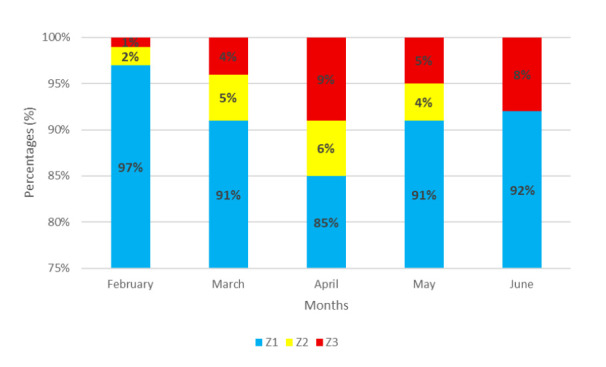
TIDv per month (%).

The swimmer followed a hybrid approach to training, incorporating both Polarized TIDv (Z1 > Z3 > Z2) and Pyramidal TIDv (Z1 > Z2 > Z3) models. A comprehensive depiction of the TIDv per month is shown in Figure 4.

### 3.4. FRPtS Volume

According to the FRPtS volume, the swimmer implemented it from March to June, with four to eight sets per month and a total volume varied from 1500 to 7900 m (Table 4).

### 3.5. FRPtS Progression

The swimmer showed significant improvement from the beginning to the last time he implemented the FRPtS. Specifically, he started the set for 200 m (8–12 × 50 m) from an average performance of 32.9 (0.47), and he finished on 29.9 (0.35) s. Additionally, the set for 400 m (2–8 × 100 m) started at 1:06.55 (1.5) min and finished at 1:00.9 (0.30) min. The RPE was stable throughout the four months, varying from 7 to 10 in both 200 and 400 m sets. The baseline performance used for the initial FRPtS was the fastest average pace he could sustain throughout the set. Following his first race participation, the FRPtS was adjusted to target a pace faster than his race performance. A depiction of the coach’s training approach and the implementation of FRPtS is provided in the Supplementary Materials (Supplementary File S1).

### 3.6. Performance Progression

The swimmer improved his PB and SB performance on the 200 and 400 m freestyle after four months. Specifically, the improvement from his PB performance was 2.9% (−3.49 s) and 1.0% (−2.55 s), whereas from his SB performance it was 2.9% (−3.53 s) and 4.4% (−11.43 s) for 200 and 400 m freestyle, respectively (Table 5).

## 4. Discussion

According to the results, after five months of training with an average monthly volume of 95,183 (45,359) m, utilizing a polarized and pyramidal training intensity distribution (TID) and completing four to eight test sets per month with total volumes ranging from 1500 to 7900 m using the FRPtS method, the MD swimmer improved both PB and SB performances in the 200 and 400 m freestyle events. The improvements relative to his PB were 2.9% and 1.0%, and relative to his SB were 2.9% and 4.4%, for the 200 and 400 m freestyle, respectively. These findings represent an initial step toward identifying modified HIIT approaches that coaches can implement as test sets to guide both themselves and their swimmers in assessing performance readiness.

### 4.1. The Conceptualization of FRPtS

The central idea behind the FRPtS construction stems from the USRPT concept and the findings of Cuenca et al. [19]. USRPT involves a high volume of repetitions over short distances, typically 25, 50, or 100 m, tailored to the swimmers’ target event. In their study, Cuenca et al. compared USRPT with an RPT protocol that differed in the prescribed swim distances (USRPT: 20 × 50 m vs. RPT: 10 × 100 m). Based on BL and RPE analyses, USRPT was associated with a lower physiological burden while yielding higher swimming speeds compared to RPT. Similar results were extracted in Papadimitriou et al. [9] study; however, the physiological load induced by USRPT does not closely replicate that of actual race conditions, considering the physiological burden between USRPT and actual events [21,22]. Consequently, the FRPtS approach was developed to address this limitation by designing a training set that more accurately reflects the physiological demands of competition while maintaining fast swimming speeds, ultimately offering a novel stimulus to enhance performance.

Considering the results, the MD swimmer improved his performance by steadily improving the FRPtS. However, the study design does not permit any generalization, because there is no clear potential of FRPtS compared to the other HIIT components of the periodization plan (i.e., RS or sprint sets). On the other hand, according to the swimmer’s RPE, FRPtS sets had an augmented burden. Moreover, he felt motivated to find a faster average pace in each set, coinciding with the performance improvement in the upcoming swimming meeting, but as reported, these conclusions can be regarded as hypotheses and not clear evidence.

### 4.2. FRPtS Volume, Intensity, and Interval

Training volume plays a pivotal role in the design of an effective swim training protocol. In the USRPT framework, Rushall [18] posits that properly structured USRPT sets are designed so that swimmers will experience failure before completing the maximum number of repetitions; this failure is not a flaw but a critical stimulus for performance enhancement. This aligns with the concept that neural fatigue and failure are central to the USRPT model, where adaptation is driven by consistently pushing the swimmer to their physiological limits [21,22]. Unlike traditional training models, which emphasize completing predetermined volumes, USRPT views failure as an essential indicator of training efficacy.

On the other hand, traditional, well-established swim programs typically prescribe fixed volumes, ranging from 50 to 6000 m, based on targeted energy system development. These sessions are generally more feasible and place less psychological and physiological strain on the athlete [24]. In the current case study, the FRPtS protocol involved training volumes of one to two times the race distance for 400 m and one to three times for 200 m, in contrast with the USPRT protocol, whose volume varies from 5 to 10 times up from the target event [22]. FRPtS volume was selected to control psychological strain while ensuring the swimmer maintained a pace faster than race speed throughout the set. Consequently, when the swimmer was not in condition to swim faster than his race pace, the set was interrupted immediately.

A critical factor for a successful training set is the balance between quantity (volume) and quality (intensity). In FRPtS, there was a controlled volume (2–3 times the distance of the event) and an intensity close to an improved performance. In all high-intensity training variations (e.g., HIIT, USRPT, SIIT, and HIFT), the physiological stimulus is significant, as indicated by markers such as HR, BL, RPE, and VO_2_max. A fundamental principle in constructing an effective training set is that its physiological load should replicate or exceed that of the target event.

In this study, FRPtS intensity was regulated solely by RPE, which is a validated and highly correlated indicator with HR [30]. The swimmer reported RPE values ranging from 7 to 9 for the 200 m set and 8 to 10 for the 400 m set, indicating a predominantly anaerobic effort. Comparable RPE values have been observed in other high-intensity swim training studies [10,31], supporting the similarity of physiological demand across these protocols. However, RPE alone cannot fully characterize training load; therefore, the results should be interpreted with caution, as additional physiological and performance indices are required to draw more definitive conclusions.

Concluding, intervals must also be tailored to the swimmer’s physiological profile and event specialization [32]. For instance, short-distance swimmers typically require longer recovery intervals than LD swimmers to achieve similar training effects [33]. When implementing a 200 m-focused FRPtS set (e.g., 12 × 50 m), a short-distance swimmer may need a near 1:1 work-to-rest ratio, whereas an LD swimmer might benefit more from shorter intervals (e.g., 20 s rest between 50 m efforts) [24]. In this case study, the swimmer’s versatility across 50 to 400 m distances justified longer intervals, particularly for the 400 m set (8 × 100 m), where approximately one minute of rest was allotted between repetitions. Additionally, the concepts of USRPT and RPT protocols were taken into account, with 20 s and 1 min intervals applied to sets of 50 and 100 m repetitions, respectively.

### 4.3. Can We Consider Its Effectiveness as a Test Set? Crucial Limitations and Future Perspectives

The present case study has several limitations that should be addressed in future research to strengthen its validity. First, the design is not appropriate for an exact safety conclusion. Interventional parallel or crossover designs would be more appropriate. Also, the intensity of each training set was determined based on the swimmer’s RPE and HR. HR measurements were taken manually by the swimmer using palpation of the carotid artery, which may reduce accuracy. Moreover, no biochemical markers were used to assess the physiological load of the training sets. Additionally, no biomechanical or kinematic analyses were conducted during the training period to evaluate technical adaptations, which may have positively influenced his performance. Lastly, it is not well established if the FRPtS is the principal component for the swimmer’s improvement. To build on these findings, future studies should incorporate crossover or parallel designs across various swimming events, involving a larger number of participants and additional physiological, biochemical, and biomechanical variables. Such research would provide a more comprehensive understanding of the effects of this type of training set on swimming performance.

## 5. Conclusions

In conclusion, the FRPtS appears to offer beneficial effects on swimming performance in MD events such as the 200 and 400 m. The FRPtS protocol involved training volumes equivalent to one to two times the race distance for the 400 m and one to three times for the 200 m. Also, the method’s burden, according to the RPE, ranged from 7 to 9 for the 200 m set and 8 to 10 for the 400 m set, indicating a predominantly anaerobic effort. Additionally, in this case study, the swimmer’s versatility across distances from 50 to 400 m justified the use of longer intervals, approximately 20 s and 1 min between repetitions for 200 and 400 m FRPtS’s, respectively. However, to better understand the effects of this type of training set on performance, additional crossover or parallel studies with a larger sample size, diverse event specializations, and comprehensive physiological and biochemical measurements are needed.

## Figures and Tables

**Table 1:** Construction of a USRPT set according to different intervals for MD and LD events. The intervals have been calculated according to the time at which the aerobic contribution has a high response.

Event (m)	Proposed Sets and Intervals During a USRPT (s)
200	20 × 25 m, @ 1:1 or 10 × 50 m, @ 1:1
400	60 × 25 m, @ 10 s or 30 × 50 m, @ 20 s or 15 × 100 m, @ 40–60 s
800	60 × 50 m, @ 5–10 s or 30 × 100 m, @ 20 s or 15 × 200 m, @ 40 s
1500	90 × 50 m, @ 5–10 s or 45 × 100 m, @ 10–15 s or 20 × 200 m, @ 20–30 s or 10 × 400 m, @ 40–50 s
10,000	100 × 100 m, @ 5–10 s or 50 × 200 m, @ 10–20 s or 25 × 400 m, @ 20–30 s or 10 × 1000 m, @ 30–40 s

@ = interval.

**Table 2:** Identification of the set’s intensity.

Zones	Zone 1a	Zone 1b	Zone 1c	Zone 2	Zone 3		Zone 4	Zone 5
Stimulus	Aerobic	Aerobic	Aerobic	Threshold	VO_2max_		LP	Sprint
					FRPtS	RS		
HR (beats/10 s)	20–22	23–24	25–26	27–28	≥29		≥29	23–24

HR: heart rate; VO_2max_: maximal oxygen consumption; FRPtS: faster race pace test set; RS: racing set; LP: lactate production.

**Table 3:** Types of sets, the time, and the reason for their implementation.

Stimulus	HR (beats/10 s)	Training Set (m)	When	Why
**Aerobic**	20–22	2–4 × 400 m @30 s	During the initial period 24 h after the Lactate set	Aerobic endurance Recovery
**Aerobic**	23–24	4–6 × 200 @20 s	During the initial period 48 h after the Lactate set	Aerobic endurance
**Aerobic**	24–26	6–8 × 100 m @15 s	During the initial period 48 h after the Lactate set Preparation for an intensive training	Aerobic endurance
**Threshold**	27–28	4–6 × 100 or 8–12 × 50 @15 or 10 s	After the initial period As a pre-lactate set	Aerobic endurance/capacity Faster muscle contraction of Type I switch
**VO_2max_**	≥29	FRPtS: 4–8 × 100 m @ 1 min or RS: 100 + 200 m all out @5 min	During the racing period i.e., one session FRPT, one RS	Aerobic capacity Aerobic capacity Lactate Tolerance Faster muscle contraction of Type I & II switches
**LP**	≥29	4–6 × 25 or 50 m @1.30 min	During the racing period i.e., one session FRPT, one RS, and one LP	Anaerobic endurance Faster muscle contraction of Type II switch
**Sprint**	23–24	6–8 × 15 or 20 m @30 s	Throughout the whole macrocycle	Alactic power Faster muscle contraction of Type II switch

VO_2max_: maximal oxygen consumption; LP: lactate production; HR: heart rate; FRPtS: faster-than-race pace test set; RS: racing set.

**Table 4:** Weeks, volume, and number of FRPtS.

FRPtS					
	February	March	April	May	June
Duration (Weeks)	−	2	4	4	1
Volume (m)	−	3000	7900	4700	1500
Practice sets for 200 m (n)		2	8	5	3
Practice sets for 400 m (n)	−	2	8	3	2

FRPtS: faster-than-race pace test set.

**Table 5:** Performance progression throughout the swimming events.

Meeting Dates	24–25 April 2021	15–16 May 2021	28–30 May 2021		9–11 June 2021 National Championship		
Events	Final heats	Final heats	Preliminaries	Finals	Preliminaries	Finals	Relay
200 m freestyle	2:00.54	1:59.02	2:00.43	2:00.07	1:57.44	1:58.18	1:57.01
400 m freestyle	4:22.91	4:17.47	4:15.65	4:15.36	4:12.34	4:11.48	−

## Data Availability

The data included in this study are available from the first author upon reasonable request.

## Supplementary Materials

The supporting information can be downloaded at https://www.mdpi.com/article/10.3390/jfmk10030291/s1, Supplementary File S1: Demonstrative depiction of the coach’s training plan.

## References

[1] Gastin P.B. (2001). Energy system interaction and relative contribution during maximal exercise. Sports Med..

[2] Duffield R., Dawson B., Goodman C. (2004). Energy system contribution to 100-m and 200-m track running events. J. Sci. Med. Sport.

[3] Papadimitriou K., Papadimitriou N., Gourgoulis V., Barkoukis V., Loupos D. (2021). Assessment of young swimmers’ technique with Tec Pa Tool. Cent. Eur. J. Sport Sci. Med..

[4] Strzala M., Stanula A., Głab G., Glodzik J., Ostrowski A., Kaca M., Nosiadek L. (2015). Shaping physiological indices, swimming technique, and their influence on 200 m breaststroke race in young swimmers. J. Sports Sci. Med..

[5] Achten J., Jeukendrup A.E. (2003). Heart rate monitoring: Applications and limitations. Sports Med..

[6] Olstad B.H., Bjørlykke V., Olstad D.S. (2019). Maximal Heart Rate for Swimmers. Sports.

[7] Nagle E.F., Nagai T., Beethe A.Z., Lovalekar M.T., Zera J.N., Connaboy C., Abt J.P., Beals K., Nindl B.C., Robertson R.J. (2019). Reliability and Validity of a Pool-Based Maximal Oxygen Uptake Test to Examine High-Intensity Short-Duration Freestyle Swimming Performance. J. Strength Cond. Res..

[8] Kabasakalis A., Nikolaidis S., Tsalis G., Mougios V. (2020). Response of Blood Biomarkers to Sprint Interval Swimming. Int. J. Sports Physiol. Perform..

[9] Papadimitriou K., Kabasakalis A., Papadopoulos A., Mavridis G., Tsalis G. (2023). Comparison of Ultra-Short Race Pace and High-Intensity Interval Training in Age Group Competitive Swimmers. Sports.

[10] Papadimitriou K., Savvoulidis S. (2020). The effects of two different HIIT resting protocols on children’s swimming efficiency and performance. Cent. Eur. J. Sports Sci. Med..

[11] Sperlich B., Zinner C., Heilemann I., Kjendlie P.-L., Holmberg H.-C., Mester J. (2010). High-Intensity Interval Training Improves VO_2_peak, Maximal Lactate Accumulation, Time Trial and Competition Performance in 9–11-Year-Old Swimmers. Eur. J. Appl. Physiol..

[12] Kilen A., Larsson T.H., Jørgensen M., Johansen L., Jørgensen S., Nordsborg N.B. (2014). Effects of 12 Weeks High-Intensity & Reduced-Volume Training in Elite Athletes. PLoS ONE.

[13] Mohr M., Nordsborg N.B., Lindenskov A., Steinholm H., Nielsen H.P., Mortensen J., Weihe P., Krustrup P. (2014). High-Intensity Intermittent Swimming Improves Cardiovascular Health Status for Women with Mild Hypertension. BioMed Res. Int..

[14] Elbe A.M., Rasmussen C.P., Nielsen G., Nordsborg N.B. (2016). High Intensity and Reduced Volume Training Attenuates Stress and Recovery Levels in Elite Swimmers. Eur. J. Sport Sci..

[15] Karabıyık H., Gülü M., Yapici H., Iscan F., Yagin F.H., Durmuş T., Gürkan O., Güler M., Ayan S., Alwhaibi R. (2023). Effects of 12 Weeks of High-, Moderate-, and Low-Volume Training on Performance Parameters in Adolescent Swimmers. Appl. Sci..

[16] Alansare A., Alford K., Lee S., Church T., Jung H.C. (2018). The Effects of High-Intensity Interval Training vs. Moderate-Intensity Continuous Training on Heart Rate Variability in Physically Inactive Adults. Int. J. Environ. Res. Public Health.

[17] Kabasakalis A., Nikolaidis S., Tsalis G., Christoulas K., Mougios V. (2019). Effects of Sprint Interval Exercise Dose and Sex on Circulating Irisin and Redox Status Markers in Adolescent Swimmers. J. Sports Sci..

[18] Rushall B.S. (2013). Understanding a USRPT Set. Swim. Sci. Bull..

[19] Cuenca-Fernández F., Boullosa D., Ruiz-Navarro J.J., Gay A., Morales-Ortíz E., López-Contreras G., Arellano R. (2021). Lower Fatigue and Faster Recovery of Ultra-Short Race Pace Swimming Training Sessions. Res. Sports Med..

[20] Williamson D., McCarthy E., Ditroilo M. (2020). Acute Physiological Responses to Ultra Short Race-Pace Training in Competitive Swimmers. J. Hum. Kinet..

[21] Papadimitriou K. (2024). Intensity and Pace Calculation of Ultra Short Race Pace Training (USRPT) in Swimming—Take-Home Messages and Statements for Swimming Coaches. Sports.

[22] Papadimitriou K. (2024). Ultra Short Race Pace Training (USRPT) in Swimming. Do the Volume and Interval Matter? A Scoping Review. Physiologia.

[23] Rodríguez F., Mader A., Chatard J.C. (2003). Energy Metabolism During 400 m and 100 m Crawl Swimming: Computer Simulation Based on Free Swimming Measurement. Biomechanics and Medicine in Swimming IX.

[24] Maglischo E.W. (2003). Swimming Fastest.

[25] Fernandes R.J., Keskinen K.L., Colaço P., Querido A.J., Machado L.J., Morais P.A., Novais D.Q., Marinho D.A., Boas J.V. (2008). Time Limit at VO_2_max Velocity in Elite Crawl Swimmers. Int. J. Sports Med..

[26] Barbosa A.C., Valadão P.F., Wilke C.F., Martins F.D.S., Silva D.C.P., Volkers S.A., Lima C.O.V., Ribeiro J.R.C., Bittencourt N.F., Barroso R. (2019). The Road to 21 Sonds: A Case Report of a 2016 Olympic Swimming Sprinter. Int. J. Sports Sci. Coach..

[27] Papadimitriou K. (2022). The Influence of Aerobic Type Exercise on Active Crohn’s Disease Patients: The Incidence of an Elite Athlete. Healthcare.

[28] Ostojic S.M., Markovic G., Calleja-Gonzalez J., Jakovljevic D.G., Vucetic V., Stojanovic M.D. (2010). Ultra Short-Term Heart Rate Recovery After Maximal Exercise in Continuous Versus Intermittent Endurance Athletes. Eur. J. Appl. Physiol..

[29] Manfredi O. (2023). Urbanchek’s Training Color System: The Palette of Swimming. SwimWarrior.

[30] Psycharakis S.G. (2011). A Longitudinal Analysis on the Validity and Reliability of Ratings of Perceived Exertion for Elite Swimmers. J. Strength Cond. Res..

[31] Arsoniadis G.G., Toubekis A.G. (2024). Progression of Sprint Interval Training Set Performance and Physiological Responses during a Six-Week Training Period. Appl. Sci..

[32] Ruiz-Navarro J.J., Santos C.C., Born D.P., López-Belmonte Ó., Cuenca-Fernández F., Sanders R.H., Arellano R. (2025). Factors Relating to Sprint Swimming Performance: A Systematic Review. Sports Med..

[33] Wiesinger H.P., Stöggl T.L., Haller N., Blumkaitis J., Strepp T., Kilzer F., Schmuttermair A., Hopkins W.G. (2025). Meta-Analyses of the Effects of High-Intensity Interval Training in Elite Athletes—Part I: Mean Effects on Various Performance Measures. Front. Physiol..

