# Supplementary material for: A Case Study on the Development of a High-Intensity Interval Training Set for a National-Level Middle-Distance Swimmer: The Conception of the Faster-than-Race Pace Test Set
Source: J Funct Morphol Kinesiol. 2025 Jul 29;10(3):291. doi: 10.3390/jfmk10030291 (PMC12371893; doi:10.3390/jfmk10030291)
Supplement: Supplementary file 1 [file jfmk-10-00291-s001.zip › jfmk-3755621-supplementary.pdf]

Supplementary File S1

Demonstrative depiction of the coach's training plan.

	February	March	April	May	June
Monday	Z1 (Aerobic) + Z3 (Sprints)	Z1 (Aerobic) + Z3 (Sprints)	Z1 (Aerobic) + Z3 (Sprints)	Z1 (Aerobic) + Z3 (Sprints)	Z1 (Aerobic) + Z3 (Sprints)
Tuesday	Z1 + Z2 (Threshold)	Z1 + Z3 (FRPtS)	Z1 + Z3 (FRPtS)	Z1 + Z3 (FRPtS)	Z1 + Z3 (FRPtS)
Wednesday	Z1 (Aerobic)	Z1 (Aerobic)	Z1 (Aerobic)	Z1 (Aerobic)	Z1 (Aerobic)
Thursday	Z1 (Aerobic) + Z3 (Sprints)	Z1 + Z2 (Threshold)	Z1 + Z2 (FRPtS)	Z1 + Z2 (FRPtS or LP)	Z1 + Z2 (FRPtS)
Friday	Z1 + Z2 (Threshold)	Z1 + Z3 (LP)	Z1 + Z3 (LP)	Z1 + Z3 (LP)	Z1 + Z3 (LP)
Saturday	Z1 (Aerobic) + Z3 (Sprints)	Z1 (Aerobic) + Z3 (Sprints)	Z1 (Aerobic) + Z3 (RS or LP)	Z1 (Aerobic) + Z3 (RS or LP)	Z1 (Aerobic) + Z3 (RS)
Sunday	OFF	OFF	OFF	OFF	OFF

LP: Lactate production; FRPtS: Faster than race pace test set; RS: Racing set.
